# Supplementary material for: Estimating the cost of implementing a facility and community score card for maternal and newborn care service delivery in a rural district in Uganda
Source: Int J Equity Health. 2021 Jan 2;20:2. doi: 10.1186/s12939-020-01335-9 (PMC7777411; doi:10.1186/s12939-020-01335-9)
Supplement: Supplementary file 3 — Additional file 3: Community score cards and citizen report cards in Uganda; What facilitates and constrains implementation? [file 12939_2020_1335_MOESM3_ESM.pdf]

Community score cards and citizen report cards in Uganda; What facilitates and constrains implementation?

Elizabeth Ekirapa Kiracho [1], Suzanne Kiwanuka [1], Noel Namuhani [1], Christine Aanyu [1], Rebecca Racheal Apolot [1]

[1] Makerere University School of Public Health

[2] Johns Hopkins University Bloomberg School of Public Health

Corresponding author

Elizabeth Ekirapa Kiracho (ekky@musph.ac.ug)

Abstract

Background; While there is some evidence about the effectiveness of social accountability initiatives using citizen or community reporting through report cards or scorecards, respectively, much less has been published about the facilitators and barriers to their implementation in low income countries. This paper aimed at identifying the facilitating factors and barriers to the implementation of community score cards (CSCs) and citizen report cards (CRCs) within the health sector in Uganda.

Methods; We carried out a qualitative study between November 2017 and Febuary 2018 among nine respondents from implementing partner organizations who had worked on CSC or CRC projects. Data collection was guided by a key informant interview guide and analysed using a thematic approach.

Results; The key factors that enhanced implementation of CSCs and CRCs included the implementing partners' provision of capacity building and continuous support to implementing teams, broad stakeholder ownership and engagement at the district and community level, problem solving mind-set across actors, advocacy and linkage with high level networks such as ministry of health Technical working groups. The factors that hindered successful implementation included community fear of reprisal by health workers and on the other hand, health worker fear of reprisal by supervisors and enlightened empowered communities, political compromise with a reduction in community voice because of the monetary incentives given to citizens by politicians, as well as a strong dependency culture among citizens, and fragmented implementation.

Conclusions; Local implementation factors such as wide scale involvement of local stakeholders, community empowerment and capacity building are essential for successful implementation of social accountability initiatives. Their wide scale implementation requires

national guidance and leadership. This would allow the development of a guiding framework for the implementation of CSCs and CRCS, subsequently enhancing integration of implementation into planning structures, work plans and budgets of relevant sectors including public agencies, civil society organizations and political actors who play a key role in accountability. Brokering partnerships between communities and stakeholders at subnational and national levels is also important for the tackling of more upstream issues identified during CSC and CRC processes.

Keywords; community scorecards, Citizen report cards, accountability, facilitators, barriers

Background

Improving the responsiveness of the health system to ensure efficient and effective delivery of health services has been an on-going challenge especially in low-and middle income countries (1, 2). It calls for the involvement of all stakeholders including health workers, political leaders, donors, and most importantly the citizens (2, 3). When empowered, citizens can influence policy, strategies and expenditure priorities at different levels of the health care system(4). Indeed individual citizens and groups can ensure that beneficiaries receive timely and quality services that address their needs and respond to their expectations by using systematic tracking of processes and activities (5). By doing this, citizens are able to hold providers accountable.

Accountability comprises of two main aspects; answerability "*the obligation of an individual or agency to provide information and make justification for their actions to other actors*" and enforcement "*the imposition of sanctions in response to the failure of power-holders to uphold their duties"*(6). From a health care standpoint, accountability is critical for reducing inappropriate use of public funds and authority, ensuring that duty bearers respect societal and professional values and standards, and augmenting service delivery and management through enhanced feedback and learning processes (7). Brinkerhoff (8) further categorizes

accountability mechanisms in the health sector as external, regulating answerability between the community and the health system and internal, focusing on bureaucratic mechanisms that regulate answerability within different levels of the health system.

Whereas these internal bureaucratic structures for accountability exist in developing countries, they are often non-functional and therefore unable to effectively hold providers accountable. Informal spaces for lodging complaints and dialogue are also similarly limited (9, 10). These weaknesses have contributed to the heightened interest in social accountability (11). Social accountability has often been defined as a type of accountability in which citizens play an active role in holding health providers and other duty bearers accountable. Social accountability tools that have been commonly used in the health sector include: citizen report cards (CRCs), participatory monitoring, budget analysis, expenditure budget tracking, social audit, public hearings, as well as CSCs (9). The CRC is a demand- side oriented tool used to solicit user feedback on service provider performance (12). The CSC is a hybrid of social accountability tools such as social audit, the citizen report card, participant defined quality and the balanced scorecard (13, 14). It is a participatory tool that has been employed to monitor and evaluate the performance of services, projects and government administrative units by the community (15).

The accountability landscape in Uganda features several mechanisms, including facility management boards, district councils, and cross-sectoral community dialogues at the sub-county level, also called "barazas". However, these structures are not consistently functional (16-18). Several organizations in the health domain have previously employed the CSC and the CRCs. These organizations have used CSCs as a tool for advocacy and to engage the community in assessing the quality of services being provided by government and non-governmental organizations. The CSC and CRC have generally been implemented in all the

four main regions of the country including; Northern, Eastern, Western and central Uganda. The Ministry of Health (MoH) has also developed several national score cards, that are used to measure achievements in addressing Reproductive, Maternal Child and Adolescent Health (RMNCAH) targets, as well as progress against the health sector development plan. The RMNCAH is a facility scorecard which mainly captures supply side issues such as number of institutional deliveries, maternal mortality rates among others (19). Unfortunately Uganda's RMNCAH score card does not capture demand side issues (such as affordability of transport to maternal health clinics, support by male partners during pregnancy and at the time of birth).

While there is some evidence about the effectiveness of the use of CSCs and CRCs, much less has been published about the facilitators and barriers to the implementation of CSCs and CRCs in low income countries (20). The existing studies do not provide a detailed account of local contextual factors and how they influence the implementation of the community score card process. Existing studies report that effective implementation of CSCs require an understanding of the local socio- political context, skilled facilitators, strong public awareness campaigns, active participation and buy-in from service providers and public agencies, implementation of locally relevant and feasible solutions, as well as coordinated and strategic follow up actions (21, 22). The barriers that were highlighted included securing and maintaining participation from different groups of stakeholders, inadequate national level advocacy and engagement(22). On the other hand, a recent systematic review on social accountability tools that included CSCs, CRCs, health facility committees and patient charters (23) identified engaging different stakeholders, use of local contextually based tools, increasing trust between citizens and leaders, increased clarity of roles, standards and responsibilities, financial and technical support from experienced groups and meaningful citizen and community involvement as the key facilitators for promoting social accountability. In addition, the review highlighted the lack of external financial and technical inputs, weak community

leadership and lack of community commitment to sustain the initiatives as factors that hindered implementation of social accountability interventions (23).

For interventions to be sustained or institutionalized, the financial, political, organizational and social environment need to be supportive and their designs need to be in synchrony with existing structures(14). The paper aims to identify barriers and facilitators to the implementation of CSCs and CRCs within the Ugandan health sector, focusing on how different CRC and CSC implementation models engage communities. These facilitators and barriers will be useful for guiding the design and implementation of initiatives aimed at promoting wide scale use of CRC and CSC initiatives that are aimed at promoting wide scale use of CSCs and CRCs in resource constrained settings.

Methods

Study design and aim,

We carried out a qualitative study between November 2017 and February 2018 with an aim of identifying facilitating factors and barriers to the implementation of community score cards (CSCs) and citizen report cards (CRCs) within the health sector in Uganda.

Study setting and participants

The study was carried out among respondents from implementing partner organizations who had implemented CSC or CRC projects in Uganda within the prevouis 10 years. We used a ten year period because we felt this would allow us to include respondents who had sufficient institutional memory to provide the answers required. Through an initial landscaping exercise on the use of CSCs we identified key institutions that had been involved in implementing CSCs and citizen report cards in Uganda, and then we identified atleast one to two individuals who had been actively involved in implementing CSCs from each of these institutions. The

institutions included UNHCO-1 interview, UNASO-1 interviews, AGHA-1, ACODE-1 interviews and UNICEF (CODES)-2 interviews, and CFI-3 interviews.

Data collection

Data collection was guided by a semi - structured interview guide that aimed to identify the experience that implementers had in implementing CSCs and CRCs, achievements obtained in the projects that they had participated in as well as facilitators and barriers to the implementation of CSCs and CRCs. Senior researchers with experience in conducting qualitative research carried out and audio-recorded all key informant interviews in English.

Data management and analysis

Research assistants transcribed the interviews verbatim. We analysed the data using a thematic approach. The explored themes related to barriers and facilitators of CSC and CRC implementation and institutionalization The transcripts were read several times by the researchers to allow them to familiarize themselves with the findings. A team of researchers that included EEK, SK, and RRA identified the codes that they expected to find in the data in terms of specific barriers and facilitators of CSC and CRC implementation and institutionalization and used this to develop the initial codebook. These included codes such as high cost of implementation, consultation of stakeholders, capacity of the facilitators. NN and EEK manually coded the transcripts and added additional codes during the coding process, as they emerged from the data. The themes emerging from the data were then identified and summarised. The list of themes was later reviewed to ensure that it covered all key issues contained in the data. Quotes illustrating meanings or key messages from the analysis were selected and some of them were presented during analysis.

Results

The principal themes that formed during the analysis included capacity building and continuous support to implementing teams, broad stakeholder ownership and engagement, problem solving mind set, advocacy and linkage with high level networks while the factors that hindered implementation included fear of reprisal, political compromise coupled with a dependency syndrome as well as fragmented implementation.

Description of the projects represented by KI's

Table 1 summarizes the characteristics of the projects from which the key informants were selected. The key informants interviewed had been involved in the implementation of projects that employed CRCs and/or community score card projects. Several of them were involved in the CODES project. This project used the lot quality assurance survey (LQAS) methodology to collect data that was presented to the community through the CRCs.The community dialogues were held to identify community problems and solutions and CODES comittees, that comprised of a team of volunteers selected by the team, were given the responsibility of ensuring that agreed upon actions were implemented. Changes in the indicators selected where captured using the CRCs and reported in subsequent dialogues. Further details about the codes project is described in the paper by Katahoire et al (24). The other key informants were involved in community score card projects which worked with local communities to identify local problems and to identify solutions to these problems. This effort was often led by a project team which worked in collaboration with community and district leaders to implement the project. Their implementation mode often followed the commonly used procedure for implementing CSCs (22).

Capacity building and continuous support to implementing teams

Almost all key informants said that building capacity of implementing teams and local technical and political stakeholders, including community members, is important for equipping

the local stakeholders with the skills required to participate in the implementation process. This would enable them to select and collect locally-relevant indicators and communicate relevant messages, hence enhancing the implementation of CSCs and CRCs. This is even more critical for the CRCs where data collection and analysis is more complicated.

Capacity building was also noted to be important for continued implementation after the end of a project. Some respondents mentioned that CSC and CRC models that rely on NGO's that are not based within the district, often do not build the capacity of local implementers adequately enough for them to continue implementing the score card activities after the exit of the project. One of the key informants pointed out that;

> *"I think what I can say is that you see it is (capacity) at organization level especially among the NGOs because they are the ones that have been largely involved in this and it is hard for us to really say with certainty that there is capacity at community level because otherwise if capacity was there why are these community initiatives not continuing where these projects have been if there was capacity then these initiatives would continue………. So the projects have rolled back with their capacity in my view back to the headquarters.* (KI Respondent 2)

It was noted that sometimes NGO's who conduct CSCs are based in the capital city and do not have offices in the districts. Such NGO's that are external to the district could still play a key role in supporting districts to sustain implement the scorecard if they work with local NGO's that are based within the districts they are supporting to implement the score card processes or if they set up a system that is embedded within the existing structures and that can be used for continued support and monitoring of the local NGO's after the project ends.

> *", if some project stops, there should be a system that can continue for some time not to stop once and leave, when you leave it also stops……. like now for us we stopped in*

December 31st the project is over staff who were at [...] dispersed, so as I talk now I don't think there is anybody, who is mindful of what is happening in the district…… so there should be some leg, ... if it is a 5-year project, at least leave a skeleton or leave like few people to continue helping the district until it is fully institutionalized" (KI Respondent 3)

One of the key informants reported that in areas where they had built capacity among the local stakeholders and local NGOs, community dialogues were being conducted even after the project had ended.

Capacity building was particularly important to ensure skilled facilitation for community meetings. Skilled facilitators who can build rapport with the community and create a safe environment for dialogue and negotiation were identified as a key ingredient for implementing CSCs and CRC's by the majority of key informants interviewed.

"*Aaah starting the whole process of getting between the communities and the duty bearers this in most cases can be hard, hard in terms of creating that constructive dialogue such that people have an open discussion, there is an initial friction that happens normally between health workers and the communities. So you need to help them overcome that process otherwise it can abort before it becomes successful so you must have a mechanism to take them through this until they reach a point where they begin to sit on the table and discuss these matters*" (KI Respondent 3)

Human, financial and non-financial resources

Human, financial and non-financial resources were also noted to be essential for a successful engagement process. The CSC processes needed to attract the involvement of influential people who had the power and authority to demand for specific action from role bearers. For example,

in one of the projects, a newly built health centre did not have lighting and one of the leaders was tasked with the responsibility of ensuring there was lighting in the health facility.

> *"a very big, beautiful maternity ward lacked lighting, so when she went in the evening ……. she got a midwife actually conducting a delivery using the torch in her small phone……. she told the sub county chief that I want electricity (in this health centre), because the electricity pole was actually inside the compound of the facility……., the sub county chief was like no no this must be a top priority for the next financial year and they now have electricity*" (KI Respondent 4)

Inadequate financial resources were one of the major barriers that was mentioned by almost all the key informants. It was reported that implementing a scorecard is very expensive due to costs such as facilitation of meetings, paying human resources and following up on agreed actions. Following up of actions is further constrained by the poor functionality of some of the structures that could be used to follow up CSCs. This poor functionality limits their usefulness in monitoring and tracking implementation of scorecard activities.

Indeed, continuous support through additional funding was pointed out as a key requirement for continued implementation by some of the respondents. It was suggested that this funding could be provided through existing budget lines, if the score card process is considered one of the normal accountability processes.

> *I think they should be institutionalised in the district plan, and investment put, because if they are institutionalised then they are budgeted for, in a way they will sort of like be sustainable, instead of getting this one NGO which pops in and the other goes and another comes in if they are institutionalised in district plans and people are taken through, how to do them it becomes part of the performance review approaches (*KI Respondent 5)

Furthermore, many projects in Uganda provide monetary facilitation for people who facilitate programs even when these programs are included in their job descriptions. This has made it difficult to sustain CSC processes in the absence of money to pay the facilitators. The community is also often facilitated for attending meetings through transport refunds and provision of drinks at the meeting. In the absence of this facilitation they also sometimes refuse to come to the dialogue meetings. Lastly half of the key informants said that implementing a scorecard is time consuming and comes along with fatigue of stakeholders. They also reported limited decision space and conditional transfer of funds as a constraint, which made it difficult to implement CSCs, since the priorities identified could not be addressed.

> "*the decision space; that is the ability of a leader to make decisions based on the local situation without much hindrance and the fiscal space; the amount of money they have to reallocate things is so thin and limited that people can't do anything about it so I think the score card is being introduced in a context in which resources are tied, so limited and tied up*" (KI Respondent 1)

Stakeholder ownership and engagement

The majority of the key informants emphasized the importance of broad stakeholder engagement and community ownership as a facilitating factor for successful implementation. Inclusion of all key stakeholders and their early involvement in the planning and implementation phases of the scorecards were reported to be essential for enhancing local ownership. Furthermore, appreciation of outputs of the scorecard process among the different stakeholders facilitates stakeholder buy in and consequently contributes to successful implementation as this arouses and maintains interest among stakeholders.

It was further stressed that making the scoring process responsive and tailored to community needs increases the participation of stakeholders in the community scorecard and citizen report

card implementation. With regard to the CRCs, it was reported that if agreed actions are not implemented, stakeholders may lose interest in continuing to analyse data for the development of the CRC and "burn out"

"*Actually for burnout I need to even qualify it, the burnout usually happens when analysis is done on some activities and nothing is done so it kills the motivation and the next time you will not be asking people to do an analysis on the same thing*"

Constructive engagement enabled different stakeholders such as politicians, health workers, and community members to express their views freely, to identify local service delivery problems and to identify what each party can do to solve the service delivery problems as explained in this quotation.

*… So beyond people asking questions they also ask themselves questions; on our part what have we done and on the part of those who should be providing services what have they done and they are able to reach a consensus on what needs to be done to improve service delivery so overall you will see uptake of services improving when these score cards are well implemented. By the way the other thing that I think people don't know with these scorecards is the fact that there is constructive engagement that occurs." (*KI Respondent 1)

It was reported that for constructive stakeholder engagement to occur, the community must be empowered. Half of the key informants mentioned that in order for the community to hold the duty bearers responsible, it is important to sensitize the community so that they know their rights, responsibilities, roles and the services that they are entitled to as well as the rights and roles of the service providers.

"The real empowerment that we talked about in this score card process must be to equip the citizens- not you as the project staff to go and ask on their behalf, it must equip them to be able to identify these problems, it must equip them to be able to engage directly with the district leadership and management on these matters…" (KI Respondent 2)

Furthermore, good leaders were considered essential for ensuring that suggestions that are identified during the dialogue meetings are actually carried out. Leadership was noted to be important for both technical and political leaders, since they are all instrumental in leading different initiatives. The importance of allowing the local stakeholders to lead the process, was explained by one of the key informants while clarifying the role that he felt implementing partners should play in the implementation process by saying;

"*…And you play more of a monitoring role, you let the district take lead otherwise when you build parallel systems you will end up suffocating the district. When you leave they will go back to their normal operations so you need to let them work within their normal operations but then twist them a bit, tap them a bit to see that…….*" (KI Respondent 6)

Advocacy and linkage with influential networks

Respondents across projects emphasized that it is critical to identify platforms and partners who can advocate for the actions agreed upon especially when these actions are beyond the control of the community. At the lower level, these platforms included for example district councils. At the ministry level it included for example technical working groups or discussions with the ministry of finance as described below.

"Our presence within the technical working groups somehow has pushed most of the health sector issues progressively towards addressing them, so in the technical working

group meetings, we usually present and say okay from our ground presence these are the issues that are at the health facility and we think these are the recommendations to address them, so issues of staffing, funding, but overall also issues of accountability" (KI -Respondent 5)

Coalitions with other CSOs were also used to put pressure and advocate for required actions. Having a common coordinated voice and relevant evidence was important for convincing the stakeholders with power that specific action was required.

The other themes that emerged were highlighted by a minority of the key informants and included lack of a problem solving mindset, fear of reprisal; political compromise coupled with a dependency syndrome as well as fragmented implementation.

Problem Solving Mind set

A few respondents also noted that successful stakeholder engagement thrives in a community that has a problem-solving mind-set so that they come to the meeting not to look for faults and blame duty bearers but rather to look for a solution. This was emphasized by one of the respondents:

"I have been telling people that come with your brains don't leave them behind because the moment you don't come with your brain this work is cumbersome it calls for you to think it calls for you to move out from the norm………. most of our people don't want to think like that they want to think like this is how we do our thing, we have always done it like this even so and so was here and we did it like this" (Respondent 9)

In the Ugandan context, some politicians have made people believe that government will provide all the requirements needed for service delivery free of charge. Communities that hold this view strongly may therefore not be willing to contribute any resources towards their health

service delivery. This dependency syndrome can therefore hinder self-help initiatives that are initiated through CSCs or CRs.

> *"I would say one thing is for sure .... they (the community) need to stop this mentality of thinking [that government is the one to solve all their problems] "government etuyambe" they need to move from that to more of what can we do for ourselves and that one does not come with a snap of a finger it requires first of all to work with them so then they have the other thing I was saying the mindset, you need to have their mind-set change from them saying government help us to I think we can do this for ourselves we can do this because you need to encourage them to contribute a certain level before they come to government*" (Respondent 8)

Fear of Reprisal

In addition to the issues listed thus far, one of the other barriers mentioned by a minority of the key informants was fear of reprisal both among duty bearers and the community members. Some of the health workers feared that they may lose their jobs if they provide the community and other role bearers with too much information, which may then encourage the community and other leaders to hold them accountable and subsequently find them at fault if they are not carrying out their expected duties. Similarly, some of the community members also feared that if they complain about the health service providers, they may be denied services.

> "*……now here a person is empowered and he feels that there is a problem, and he feels like taking the initiative to push for change but now here is a poor lady who gets services from this very health facility, which is a public health facility where they expect relatively free services, and now when she goes there and reports, to either sub county or to any other official, there was that fear that once am identified, that once, a person*

comes to realize it's me, I will be denied services and I don't have another alternative, because I can't afford a private health facility, that was one." (KI Respondent 6)

Political Compromise

Political compromise was also highlighted by one key informant as a barrier. In the Ugandan context politicians often give citizens money and other basic requirements such as sugar, hoes etc. in exchange for their votes. Subsequently, some community members find it hard to hold accountable the same person who gave them these gifts.

"[...] there was also another issue of political compromise during elections, these are the people who give out, material things during elections like soap, salt, he is a leader, he is a chairperson LC2, LC3, LC5, now at the end of the day, you realize that actually most of the issues that are happening at the health facility, for example at the lower level health facilities, HC2, HC3, is because there is inadequate monitoring and supervision and who monitors? It's the politicians, so which means it is the politician, who is accountable and who should be accountable, but how will the person, who received some material gifts from the person he is trying to hold accountable [take action], will he or she really hold her/him accountable?...." (KI-Respondent 7)

Fragmented Implementation

Two of the key informants who are affiliated with academic institutions held the view that the role of CSCs within the health sector has not been clearly stipulated or communicated to key implementers and the public. Secondly, they felt that there is no standard way of implementing CSCs hence different implementers have used different models and different methods to implement CSCs. This has therefore led to fragmented implementation of the CSCs in the country with a multiplicity of scorecards that are not harmonized.

Discussion

Social accountability initiatives such as CSCs and CRCs have been present in Uganda for a long time, yet usually remained local and at a small-scale. Our exploration seeks to understand the factors that could enhance the implementation of such social accountability interventions, in hopes that these could inform future initiatives. Our work identified the following key factors that could enhance implementation of CSCs capacity building and continuous support to implementing teams, broad stakeholder ownership and engagement, problem solving mind-set, advocacy and linkage with high level networks. On the other hand, the factors that hindered implementation included fear of reprisal, political compromise coupled with a dependency syndrome as well as fragmented implementation.

National leadership in guiding implementation of CSCs and CRCs is critical for their successful implementation and for implementation at scale. Although CSCs and CRCs have been implemented in Uganda over the past two decades, there is still no wide scale application of these tools within the health sector. Most of the community score card projects in Uganda, have been implemented by NGOs. Whereas implementation that is largely led by NGOs was successful in achieving the desired objectives (24), often the NGOs did not leave in place a system that could support continued implementation. This has subsequently led to a situation where there is a plethora of fragmented implementation efforts by different NGOs implementing different types of score cards using different methods, inadequate capacity building of local stakeholders to implement the score cards and subsequently lack of continuity of implementation of the score cards after the project exits.

Firstly, the national leadership needs to provide a guiding framework for the implementation of CSCs and CRCs. This would include identifying key features of the scorecard or citizen report card that should be implemented nationally by districts and partners who can further adopt it. Secondly it needs to provide a guiding framework for the implementation process.

This would include identifying key structures that are to get involved in the implementation of CSCs. The capacity of the local stakeholders and other non-state actors to implement CSCs should inform the implementation model employed as well as selection of structures that are to be used. Inclusion of government structures can give the CSCs and CRC's the stability and legitimacy that is required for its continued implementation, while inclusion of non-state actors provides the objectivity that is essential in social accountability. Work by Wild showed that in some contexts it would be futile to insist on working outside or against the public agencies, and some degree of alliance often yielded more positive results compared to none at all (21). Furthermore, if wide scale implementation is desired predictability of donor funding on which many non-state agencies depend and nationwide presence of these agencies especially in more rural areas needs to be carefully considered.

Thirdly it needs to guide allocation of resources that can be used for wide scale implementation and for investment in building the capacity of these institutions. This could also allow government to coordinate resources from NGOs involved in implementing CSCs allowing for their equitable distribution throughout the country and systematic long term investment in capacity building initiatives meant to strengthen and support implementation at local level. This kind of capacity building should eventually equip the community and its leaders with the skills necessary to support and to push for inclusive service delivery in their communities on their own.

Fourthly, government can provide a framework for its integration into planning structures, work plans and budgets of relevant sectors including government agencies, NGO oversight structures and political actors who play a role in accountability. This would make it easier to feed issues identified during the score card implementation into management and decision-making platforms at subnational and national level. Such decision-making platforms would

then have the responsibility of addressing the problems identified. This would create a conducive environment for wide scale implementation of the community and citizen report cards.

While government needs to play a central role in guiding implementation, the importance of local stakeholder ownership and engagement during implementation of community scorecard and citizen report card processes cannot be over emphasized. It aids active participation of community, political and technical stakeholders which is critical in facilitating implementation of the score card and achieving the desired objectives (9, 22-24). This also allows communities to participate more actively in improving their health as co producers of their health(9) . Lastly stakeholder ownership and engagement also promotes the formation of partnerships and coalitions that have been noted to increase achievement of agreed outputs (25). A deliberate effort therefore needs to be made to involve key local technical and non-technical leaders who have the power to initiate the actions required to bring about desired changes. In Ghana, involvement of local chiefs led to more successful implementation of a maternal and new-born CSC project (26). There is evidence that the effectiveness of local leaders in holding providers accountable is sometimes hindered by their limited knowledge about the rights and responsibilities of communities, providers and different role bearers or by lack of clarity about their roles(5, 22, 23). Community score card processes have been shown to be instrumental in empowering local leaders with the information that they need, as well as clarity about their roles with regard to accountability(9, 23, 26). Additionally, in some cases the problems identified during the community engagement process can not be solved locally. They need the involvement of highly placed officials who belong to networks that can influence upstream issues or highly politicized issues. Brokering of partnerships and linkage with networks that are influential enough to bring about desired changes at the local or national level come to play in such situations (21).

Social accountability approaches are hinged around the active involvement of citizens, their adequate preparation before implementation starts is therefore of paramount importance. This preparation should include both sensitization about the CSC and CRC implementation process and community empowerment. In the absence of this, they may have expectations of providers that are not aligned with the roles and rights of the providers (22, 23). Such misunderstandings can lead to conflicts between the community and the health workers. On the other hand, some of the communities may not be aware that they are supposed to provide oversight and can therefore hold providers accountable(10, 24) especially in an "*environment where providers are seen as demi gods who hold the keys to good health"* Furthermore, they may fear to voice their complaints, thinking that they will be denied services or will be mistreated if they complain about the services provided to them (22). Additionally, implementers need to be aware of the power dynamics that often manifests through fear of reprisal, political compromise and a dependency syndrome that can dampen efforts to get communities actively involved in solving health service delivery and utilization problems (10). Skilled facilitators and buy in and active participation of influential local leaders becomes very important in neutralizing such negative influences. Engagement of political leaders and community leaders early is important for getting them on board so that they can support such initiatives that encourage the community to take charge of their health and local problems. Their early involvement in the CSCs and presentation of community score card objectives in a manner that allows them to appreciate the usefulness of the score card can be instrumental in getting their support. Lastly, our work brought to light the importance of the community having a problem-solving mind-set. This kind of mind-set creates an enabling environment for constructive engagement and for the generation of local initiatives that could solve local problems identified through the CSC and CRC process.

Study limitations and strengths; Since this is a purely qualitative study it was not possible to accurately quantify the extent to which the different factors facilitate or constrain implementation. Recall bias may also have influenced the findings since some respondents had implemented the projects some years back. However the kind of information we collected is unlikely to have been heavily influenced by recall. Furthermore we interviewed several key informants to allow us to get a wide variety of experience and so we are confident that we captured the key issues. Although all the experiences reported are from work done in Uganda we believe that the findings are generalizable to other low and middle income countries.

Conclusions

Although CSCs and CRCs have been piloted in many countries few countries have scaled them up nationwide. According to our findings some of the key positive influencers of implementation included capacity building and continuous support to implementing teams, broad stakeholder ownership and engagement, having a problem-solving mind-set, advocacy and linkage with high level networks. On the other hand, negative influencers included fear of reprisal, political compromise coupled with a dependency syndrome as well as fragmented implementation. Additionally, our work high lights the key role that national governments can play in guiding wide scale implementation through developing a guiding framework for the implementation of CSCs and CRS and ensuring integration of implementation into planning structures, work plans and budgets of relevant sectors including public agencies, NGO oversight structures and political actors who play a key role in accountability. This is likely to promote coordinated implementation and leveraging of resources that exist among non-state actors so as to allow long term systematic funding and investment of the funds into building local capacity and support for continued implementation of community and CRCs.

Brokering partnerships at subnational and national level is also important for the tackling of more upstream issues. This calls for deliberate efforts to engage and link with influential people

from the onset of the process. Lastly power dynamics that manifest through fear of reprisal, political compromise and a dependency syndrome need to be countered through community empowerment and early engagement of influential actors who can counteract such influences and instead promote the development of a problem-solving mind-set in the community that can heighten community efforts to improve health.

List of Abbreviations

CSC	Community Scorecard
CRC	Citizen Report card
ACODE	Advocates Coalition for Development and Environment
NGO	Non-Governmental Organization
LC	Local council
HC	Health centre
AGHA	Action Group for Health, Human Rights and HIV/AIDS
CSOs	Civil society organizations
UNHCO	Uganda National Health consumers Organization
UNASO	Uganda Network AIDS Service Organisations
UNICEF	United Nations Children's Fund
CFI Child	Fund International
RMNCAH	Reproductive maternal Newborn child and Adolescent Health

Declarations

Ethical approval and consent to participate

Ethical clearance for the study was obtained from the Makerere University School of Public Health Higher Degrees, Research and Ethics Committee (MakSPH HDREC) and the Uganda National Council of Science and Technology (UNCST) (study number SS 4323). The

objectives, benefits and risks of the study were explained to the study participants and written informed consent obtained from all the study participants. All data obtained during the study were treated as confidential and anonymous identifiers were used. We restricted data access to only the investigators and the research assistants.

Consent for publication

Consent to publish the findings of the study was obtained at the point of seeking consent to participate. The participants' confidentiality and anonymity while reporting was assured.

Availability of data and materials

The data used to undertake this study is safely stored by the monitoring and evaluation team of the FHS study at Makerere University School of Public Health.

Competing interests

We declare no conflict of interest

Funding

The study was funded by the Future Health Systems Consortium (FHS), through a grant from DFID.

Authors' contributions

All authors contributed towards the conceptualization of the paper. EKK and NN and RRA led the drafting of the manuscript. NN did the data coding and EEK conducted the analysis for the study. All authors reviewed the drafts, provided significant intellectual input and approved the final manuscript.

Acknowledgements

Special thanks to DFID who provided funding for this paper through the Future Health Systems consortium. We also appreciate the key informants who provided data for this paper.

References

1. Bhargava V. Strategies for Empowering Communities to Demand Good Governance and Seek Increased Effectiveness of Public Service Delivery. Partnership For Transparency Fund. Partnership for Transparency Fund. 2012.

2. Mirzoev T, Kane S. What is health systems responsiveness? Review of existing knowledge and proposed conceptual framework. BMJ global health. 2017;2(4):e000486.

3. Ackerman JM. Social accountability in the public sector: a conceptual discussion. Social development papers. 2005;82:1-44.

4. Church J, Saunders D, Wanke M, Pong R, Spooner C, Dorgan M. Citizen participation in health decision-making: past experience and future prospects. Journal of public health policy. 2002;23(1):12-32.

5. Edward A, Osei-Bonsu K, Branchini C, shah Yarghal T, Arwal SH, Naeem AJ. Enhancing governance and health system accountability for people centered healthcare: an exploratory study of community scorecards in Afghanistan. BMC health services research. 2015;15(1):299.

6. Schedler A, Diamond LJ, Plattner MF. The self-restraining state: power and accountability in new democracies: Lynne Rienner Publishers; 1999.

7. Edward A, Zare H, Malama S, Bowles C. ISQUA17-3007 advancing patient centered care through social audit mechanisms in rural zambia: evidence on pediatric quality of care. international journal for quality in health care. 2017;29(1):21-2.

8. Brinkerhoff D. Accountability and health systems: overview framework and strategies. 2003. Abt Associates Inc. https://www.who.int/.../accountability/AccountabilityHealthSystemsOverview.pdf

9. Ho LS, Labrecque G, Batonon I, Salsi V, Ratnayake R. Effects of a community scorecard on improving the local health system in Eastern Democratic Republic of Congo:

qualitative evidence using the most significant change technique. Conflict and health. 2015;9(1):27.

10. de Wit JJ, Akinyoade AA. Accountability in formal and informal institutions: a cross country analysis. ISS Working Paper Series/General Series. 2008;464:1-35.

11. Yimenu EB. Social Accountability Mechanisms in enhancing good governance New york: 2011.
https://www.un.org/en/conf/ldc/pdf/eshetu%20b.pdf

12. Waglé S, Singh J, Shah P. Citizen Report Card Surveys: A Note on the Concept and Methodology. 2004.

13. Peters DH, Noor AA, Singh LP, Kakar FK, Hansen PM, Burnham G. A balanced scorecard for health services in Afghanistan. Bulletin of the World Health Organization. 2007;85:146-51.

14. Bisbe J, Barrubés J. The balanced scorecard as a management tool for assessing and monitoring strategy implementation in health care organizations. Revista Española de Cardiología (English Edition). 2012;65(10):919-27.

15. Rabbani F, Lalji SN, Abbas F, Jafri SW, Razzak JA, Nabi N, et al. Understanding the context of balanced scorecard implementation: a hospital-based case study in Pakistan. Implementation Science. 2011;6(1):31.

16. UNHCO M. Client Satisfaction with Services in Uganda's Public Health Facilities A Study by the Medicines Transparency Alliance (MeTA), Uganda. 2013.

17. Green C. Community monitoring in a volunteer health worker setting: a review of the literature. Kampala, Uganda: inSCALE-Malaria Consortium. 2011.

18. Development Research and Training. Do citizens' Views matter in planning and budgeting in Uganda Internet 2014. Available from: http://www.drt-ug.org/wp-content/uploads/2016/10/PPA-planning-and-budgeting-brief1.pdf.

19. UNHCO. Uganda's Maternal Health Score Card to enhance transparency and accountability Uganda: Uganda National Health consumer Organization 2016 [cited 2018 04. May. 2018]. Available from: http://unhco.or.ug/ugandas-maternal-health-score-card-to-enhance-transparency-and-accountability/.

20. GSDRC. Helpdesk Research Report: Evaluations of Voice and Accountability Instruments. 2010. www.gsdrc.org/docs/open/gsdrc_vea_topic_guide.pdf

21. Wild L, Wales J, Chambers V. CARE's experience with community score cards: what works and why. London: Overseas Development Institute. 2015.

22. Gullo S, Galavotti C, Altman L. A review of CARE's Community Score Card experience and evidence. Health policy and planning. 2016;31(10):1467-78.

23. Danhoundo G, Nasiri K, Wiktorowicz ME. Improving social accountability processes in the health sector in sub-Saharan Africa: a systematic review. BMC public health. 2018;18(1):497.

24. Katahoire AR, Henriksson DK, Ssegujja E, Waiswa P, Ayebare F, Bagenda D, et al. Improving child survival through a district management strengthening and community empowerment intervention: early implementation experiences from Uganda. BMC public health. 2015;15(1):797.

25. ten Hoope-Bender P, Martin Hilber A, Nove A, Bandali S, Nam S, Armstrong C, et al. Using advocacy and data to strengthen political accountability in maternal and newborn health in Africa. International Journal of Gynecology & Obstetrics. 2016;135(3):358-64.

26. Blake C, Annorbah-Sarpei NA, Bailey C, Ismaila Y, Deganus S, Bosomprah S, et al. Scorecards and social accountability for improved maternal and newborn health services: A pilot in the Ashanti and Volta regions of Ghana. International Journal of Gynecology & Obstetrics. 2016;135(3):372-9.
